# Determination of the optimal concentration and duration of C5aR antagonist application in an inflammatory model of human dental pulp cells

**DOI:** 10.1002/2211-5463.13571

**Published:** 2023-02-20

**Authors:** Junlong Hu, Xiaohan Tan, Xiaoling Wei, Weiping Hu, Li Gao, Xiaofang Cao, Huiying Yang, Zhuling Jiang, Ning Li, Li Teng, Mingyue Liu

**Affiliations:** ^1^ Department of Craniomaxillofacial Surgery, Plastic Surgery Hospital Chinese Academy of Medical Sciences and Peking Union Medical College Beijing China; ^2^ Department of Prosthodontics The second Affiliated Hospital of Harbin Medical University China; ^3^ Hainan Dental Hospital Haikou China; ^4^ Department of Oral and Maxillofacial Surgery The second Affiliated Hospital of Harbin Medical University China; ^5^ Department of Endodontics The second Affiliated Hospital of Harbin Medical University China; ^6^ Department of Stomatology Qiqihar Eye & ENT Hospital China; ^7^ Department of Oral Implantology The second Affiliated Hospital of Harbin Medical University China; ^8^ Department of Cardiology The second Affiliated Hospital of Harbin Medical University China; ^9^ Department of Prosthodontics The second Affiliated Hospital of Harbin Medical University & The Key Laboratory of Myocardial Ischemia Ministry of Education China

**Keywords:** C5aR, C5aR antagonist W54011, dental pulp cells, interleukin‐6, lipopolysaccharides, tooth decay

## Abstract

Deep tooth decay approaching the pulp may develop into pulpitis; to prevent this, pulp cells need to balance the rapid immune response to avoid rapid swelling of the pulp. Current treatment of deep decay that approaches the pulp involves the application of drugs that induce low‐level inflammation in the dental pulp to promote its repair, but this treatment is sometimes insufficient. However, the unsuccessful treatment often resulted in pulpitis. The C5a‐C5aR is the initial stage of the immune cascade response. Blocking the binding of C5a‐C5aR can slow the immune response in the narrow pulp cavity, so that dental pulp cells have enough time to proliferate, migrate, and differentiate. In this study, we compared lipoteichoic acid (LTA) and lipopolysaccharides (LPS) at different concentrations and time points and used the C5aR antagonist W54011 to block the C5a‐C5aR axis. The blocking effect was detected by analyzing the expression of *C5a*, *C5aR*, interleukin (IL)‐6, and Toll‐like receptors 2 and 4 (*TLR‐2, 4)*. Next, we determined the optimal concentration and duration of LTA and LPS treatment in combination with W54011. Based on our results, we selected 1.0 μg·mL^−1^ LPS treatment for 48 h to generate an inflammatory model of human dental pulp cells. We then regrouped the cells and conducted expression analyses to monitor the expression of C5a, C5aR, IL‐6, and TLR‐4 at the protein and mRNA levels. LPS stimulation for 48 h and treatment with W54011 for 48 h effectively inhibited inflammation and did not affect C5a expression. This study provides a basis for follow‐up studies of W54011 in dental pulp cells.

## Graphical abstract

The key mechanism of successful pulp preservation is the balance between immunity and regeneration. C5a binds to its receptor and plays the strongest role in dental pulp immunity. To block the interaction between C5a and C5aR by using C5aR antagonists can downregulate the expression of IL‐6, which helps to slow down the immune process, which in turn can facilitate pulp repair.

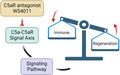

**Abbreviations**
CCK‐8: cell counting Kit‐8
DMEM: Dulbecco's modified Eagle's medium
IL‐6: interleukin 6
LPS: lipopolysaccharides
LTA: lipoteichoic acid
MAPK: mitogen‐activated protein kinase
PBS: phosphate‐buffered saline
TLR: Toll‐like receptor

Clinically, the treatment of deep decay approaching the pulp involves the application of soothing materials that induce low‐level inflammation in the dental pulp to promote its repair. The expression of inflammatory factors can amplify the immune cascade and play an important role in the immune response. The dental pulp lies within a relatively closed pulp cavity, and consequently, has poor metabolic circulation. Hence, when the dental pulp becomes congested, it will not have adequate space to swell like skin tissues, to metabolize edema and other stress reactions, and can therefore lead to pulpitis. Therefore, effectively controlling the degree of the immune response in the dental pulp, to facilitate pulp repair, is necessary to prevent pulpitis. A key mechanism for successful pulp preservation lies in the balance between immunity and regeneration. When deep caries near the pulp occurs, the dental pulp is irritated, a nonspecific immune response occurs in the dental pulp, and the complement system is activated. This system comprises a network of proteins that play an important role in defense and inflammation. Among these proteins, the anaphylatoxins are a group of pro‐inflammatory polypeptides that promote immune progress and include C3a, C4a, and C5a. The expression of C5a is 25 and 2500 times that of C3a and C4a, respectively. Complement C5a‐C5aR interaction enhances mitogen‐activated protein kinase (MAPK) signaling activities to mediate renal injury in trichloroethylene‐sensitized BALB/c mice [1]. C5a plays the strongest role in chemotactic immune cells and is central to dental pulp immunity [2, 3]. Therefore, inhibiting the C5‐C5a receptor axis is a good way to balance immunity [4]. Therefore, we hypothesized that blocking the interaction between C5a and C5aR can downregulate the expression of interleukin (IL)‐6, which helps to slow down the immune process.

Lipoteichoic acid (LTA) and lipopolysaccharides (LPS) can be used to generate an inflammatory model of human dental pulp cells (HDPCs) *in vitro* [5]. LTA binds to the plasma membrane of Gram‐positive bacteria and is recognized by Toll‐like receptor 2 (TLR‐2). LPS is the major component of the outer membrane of Gram‐negative bacteria, with the Toll‐like receptor 4 (TLR‐4) involved in its recognition and signal initiation. Studies have confirmed that in cases of shallow caries, Gram‐positive bacteria account for a higher proportion, while in cases of deep caries and pulpitis, the proportion of Gram‐negative bacteria is higher [6]. Previous studies reported that both LTA and LPS were used as an inflammation model for dental pulp cells [7, 8, 9, 10]. Our previous studies compared dental pulp cell inflammation models constructed using LTA and LPS, and the different treatment times with LTA and LPS (1, 2, 3, 5 and 7 days) [5]. We showed that LPS had a stronger effect on promoting the expression of inflammatory factor interleukin (IL)‐6 in dental pulp cells. Moreover, we showed that 48‐h LTA and LPS stimulation was the best choice for creating dental pulp cell inflammation models [5]. This is compared to a stimulation time of 2 h employed by most studies [11, 12]. However, no definitive comparative studies exist on the duration of LTA and LPS action. Therefore, this study will further compare the stimulatory effects of LTA and LPS stimulation for 48 h and 2 h based on the expression of C5aR, IL‐6, TLR‐2, and TLR‐4.

## Materials and methods

### Isolation and culture of human dental pulp cells

Human dental pulp cells were prepared from immature third molars at the 2/3 root formation stage using the explant outgrowth method [13]. Teeth were obtained from at least three different donors per experiment (*n* = 12; 4 molars per donor; age = 18–25 years; 1 : 1 male to female). Surgeries were performed at the Oral and Maxillofacial Surgery Department of the Second Affiliated Hospital of Harbin Medical University (Harbin, China). The present study was performed following the guidelines set forth by the Declaration of Helsinki and approved by the Institutional Ethics Committee of the Second Affiliated Hospital of Harbin Medical University, under the ID number KY2020‐57. Written informed consent was obtained from all patients. The basic cell culture medium consisted of Dulbecco's modified Eagle's medium (DMEM)/F‐12 (Hyclone; GE Healthcare Life Sciences, Logan, UT, USA), supplemented with 15% fetal bovine serum (Biological Industries, Kibbutz Beit Haemek, Israel), 1% penicillin, and 1% streptomycin.

### Cell groups and treatment conditions

Third‐generation pulp cells (8 × 10^4^) were cultured in 100 mm dishes in a 37 °C incubator with 5% CO_2_. The cells were washed thrice with phosphate‐buffered saline (PBS) and incubated in serum‐free DMEM (6 mL per well) for 24 h. Next, the cells were cultured in serum‐free DMEM containing 1% penicillin and 1% streptomycin, and divided into 11 groups (Table 1) for stimulant treatments as follows: (a) 1.0 μg·mL^−1^ LTA for 48 h followed by 1.0 μg·mL^−1^ W54011 (HY‐16992A; MedChemExpress, Meddlesex, NJ, USA) for 72 h; (b) 1.0 μg·mL^−1^ LPS for 48 h [14] followed by 1.0 μg·mL^−1^ W54011 for 48 h; (c) 1.0 μg·mL^−1^ LPS for 48 h followed by 1.0 μg·mL^−1^ W54011 for 72 h; (d) 1.0 μg·mL^−1^ LTA for 48 h followed by DMEM for 72 h; (e) 1.0 μg·mL^−1^ LPS for 48 h followed by DMEM for 72 h; (f) 10 μg·mL^−1^ LTA for 2 h followed by 10 μg·mL^−1^ W54011 for 2 h; (g) 10 μg·mL^−1^ LPS for 2 h followed by 10 μg·mL^−1^ W54011 for 2 h; (h) 10 μg·mL^−1^ LTA for 2 h [15]; (i) 10 μg·mL^−1^ LPS for 2 h; (j) DMEM alone for 2 h; (k) no stimulus (control group).

**Table 1:** Grouping of different stimuli concentrations. W/O, without.

Group	LTA	LPS	W54011	DMEM
1	1.0 μg·mL^−1^, 48 h	W/O	1.0 μg·mL^−1^, 72 h	W/O
2	W/O	1.0 μg·mL^−1^, 48 h	1.0 μg·mL^−1^, 48 h	W/O
3	W/O	1.0 μg·mL^−1^, 48 h	1.0 μg·mL^−1^, 72 h	W/O
4	1.0 μg·mL^−1^, 48 h	W/O	W/O	72 h
5	W/O	1.0 μg·mL^−1^, 48 h	W/O	72 h
6	10 μg·mL^−1^, 2 h	W/O	10 μg·mL^−1^, 2 h	W/O
7	W/O	10 μg·mL^−1^, 2 h	10 μg·mL^−1^, 2 h	W/O
8	10 μg·mL^−1^, 2 h	W/O	W/O	W/O
9	W/O	10 μg·mL^−1^, 2 h	W/O	W/O
10	W/O	W/O	W/O	2 h
11	W/O	W/O	W/O	W/O

### Cell proliferation and cytotoxicity assays

The Cell Counting Kit‐8 (CCK‐8) was used to assay cell proliferation and cytotoxicity. According to their grouping, the cells were seeded in 96‐well plates (5 × 10^3^ cells per well), with each group having two sets of secondary wells. Cells were divided into the following groups: (a) serum‐free DMEM containing 1.0 μg·mL^−1^ W54011 lacking cells; (b) 20% FBS culture medium; (c) serum‐free DMEM as the control group; (d) serum‐free DMEM containing 0.5 μg·mL^−1^ W54011; (e) serum‐free DMEM containing 1.0 μg·mL^−1^ W54011; (f) serum‐free DMEM containing 1.5 μg·mL^−1^ W54011; (g) serum‐free DMEM containing 1 μL DMSO. All six groups were cultured in a 37 °C, 5% CO_2_ cell incubator for 24 and 48 h, respectively. Next, 10 μL of the CCK‐8 reagent was added, and the absorbance of each well was measured at 450 nm by an automatic enzyme plate analyzer after 2 and 4 h in the incubator.

### Reverse transcription‐polymerase chain reaction (RT‐PCR)

Total RNA was extracted using the RNA prep pure Cell/Bacteria Kit (TIANGEN, Beijing, China) according to the manufacturer's instructions. Complementary DNA was synthesized from the RNA using a ReverTra Ace™ qPCR RT Kit (TOYOBO, OSAKA, Japan) using the following cycling conditions: cDNA synthesis, 65 °C for 5 min and 37 °C for 15 min; 98 °C for 5 min; PCR: 95 °C for 10 min (initial denaturation), 40 cycles of 95.0 °C for 15 s, 58 °C for 30 s, 5 s at 70–90 °C at increments of 0.5 °C. The primer sequences used are presented in Table 2. imagej (NIH, MD, USA) was used to quantify the intensity of the amplicons on the agarose gel.

**Table 2:** Primers sequences used for RT‐PCR.

Gene	Forward primer (5′–3′)	Reverse primer (5′–3′)
*5a*	CGTTTGTCGTGGCTGTAGTCC	GACCGCTTTCTGCTGGTGTTT
*C5aR*	CGCTTTCTGCTGGTGTTTA	GTTTGTCGTGGCTGTAGTCC
*TLR‐4*	ACTGGGTAAGGAATGAGC	CTGGGACACCACAACAAT
*IL‐6*	GAGGAGACTTGCCTGGTG	GCATTTGTGGTTGGGTCA
*TLR‐2*	ATCCTCCAATCAGGCTTCTCT	GGACAGGTCAAGGCTTTTTACA
*Actin*	AAGGAGCCCCACGAGAAAAAT	ACCGAACTTGCATTGATTCCAG
*C5aR*	TCCTTCAATTATACCACCCCTGA	ACGCAGCGTGTTAGAAGTTTTAT

### Cell groups and culture conditions

Based on the RT‐PCR results of the stimulant treatments and treatment times, the cells were regrouped into six groups as follows: (a) 1.0 μg·mL^−1^ LPS for 48 h followed by 1.0 μg·mL^−1^ W54011 for 48 h; (b) 1.0 μg·mL^−1^ LPS for 48 h followed by 1.0 μg·mL^−1^ W54011 for 72 h; (c) serum‐free DMEM alone for 4 days; (d) normal culture medium for 4 days (negative control); (e) 1.0 μg·mL^−1^ LPS for 48 h followed by serum‐free DMEM for 48 h; (f) serum‐free DMEM for 48 h followed by 1.0 μg·mL^−1^ W54011 for 48 h. Prior to each treatment, the cells were starved without FBS for 24 h, divided into six groups, and incubated in serum‐free DMEM (6 mL per well) for 24 h.

### Quantitative RT‐PCR (RT‐qPCR)

Gene expression was quantified using the FastStart Universal SYBR‐Green Master Mix (Roche, IN, USA). The RT‐qPCR cycling conditions were as follows: 95 °C for 10 min (initial denaturation), 40 cycles of 95 °C for 30 s, 55 °C for 30 s, 72 °C for 30 s, and 72 °C for 5 min. Target gene expression was normalized against the reference gene *Actin* to correct for nonspecific experimental variation. The 2^−ΔΔCt^ method (Livak method) [16] was used to determine the relative expression. The primers listed in Table 1 were used for RT‐qPCR.

### Western blotting

Total cellular proteins were extracted from the cultured cells using a cell lysis buffer (Beyotime, Shanghai, China), consisting of 150 mm NaCl, 20 mm Tris (pH 7.5), 2.5 mm sodium pyrophosphate, 1% Triton X‐100, 1% (w/v) Na_3_CO_4_, 0.5 μg·mL^−1^ leupeptin, 1 mm EDTA, and 1 mm phenylmethanesulfonylfluoride (PMSF). Total protein was quantified using the Pierce™ BCA Protein Assay kit according to the manufacturer's protocol (Thermo Fisher Scientific, Inc., Shanghai, China). Twenty micrograms of each sample were separated on a 10–15% SDS/PAGE gel. Separated proteins were transferred onto PVDF membranes (Bio‐Rad Laboratories, Inc., Hercules, CA, USA) and blocked with 5% (w/v) milk at room temperature for 1 h with gentle agitation. Next, the membranes were washed by PBST and blocked by in 1% BSA with 0.05% Tween‐20 at 4 °C overnight with the following primary antibodies (purchased from ProteinTech Group, Inc., Rosemont, IL, USA): anti‐C5aR rabbit polyclonal antibody (cat. No. 21316‐1‐AP; 1 : 1000), anti‐IL‐6 (cat. No. 66146‐1‐lg; 1 : 1000), anti‐TLR‐4 (cat. No. 19811‐1‐AP; 1 : 1000), anti‐β‐actin (cat. No. 20536‐1‐AP; 1 : 5000). The following day, the membranes were incubated with the following secondary antibodies (purchased from ProteinTech Group, Inc.) at room temperature for 1 h: goat anti‐mouse IgG (cat. No. SA00001‐1; 1 : 5000) and goat anti‐rabbit IgG (cat. No. SA00001‐2; 1 : 10 000). The Blue Plus® IV Protein Marker (10–180 kDa, cat. No. DM131‐01, TransGen Biotech, Beijing, China) was used to monitor electrophoresis and membrane transfer. Bands were visualized using the Ultrasensitive ECL Detection Kit (PK10003, ProteinTech Group, Inc.) and photographed using the Tanon 1000 digital image gel analytical system (Tanon Science & Technology Co., Ltd., Shanghai, China). β‐Actin was used as the loading control. The bands were quantified using image j.

## Results

### Cell morphology and proliferation of dental pulp cells

**Fig. 1:**
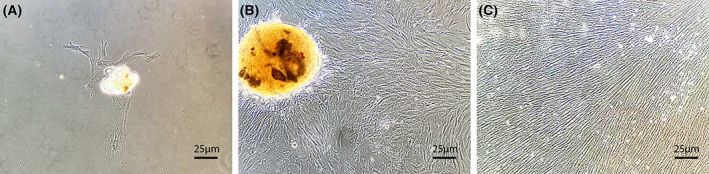
Morphological appearance of dental pulp cells (Original magnification, ×40). (A) The appearance of dental pulp cells at day 4 of culture, under the microscope, there is adherent growth of dental pulp cells. (B) Cells started to form an aggregated center with increased extracellular matrix deposition. Generate a large clonogenic cell population after 2 week‐culture, dental pulp cells proliferate as a typical fibroblast‐like morphology. (C) Proliferation of DPSCs enters an active stage after passaging, format the hill‐and‐valley organization. The length of the scale bars is 25 μm.

Primary culture human dental pulp cells adhered to the wall of the culture flask on day four (Fig. 1A) and generated clonogenic cell populations by day seven. Cells were spindle‐shaped with typical fibroblast‐like morphology. After 14 days, cell morphology was more uniform, with a fibroblast morphology and long cell processes arranged in parallel and reaching a sufficient number for passage (Fig. 1B). After passage, the cells proliferated rapidly (Fig. 1C).

### A concentration of 1.0 μg·mL^−1^ W54011 was selected following the CCK‐8 assay

**Fig. 2:**
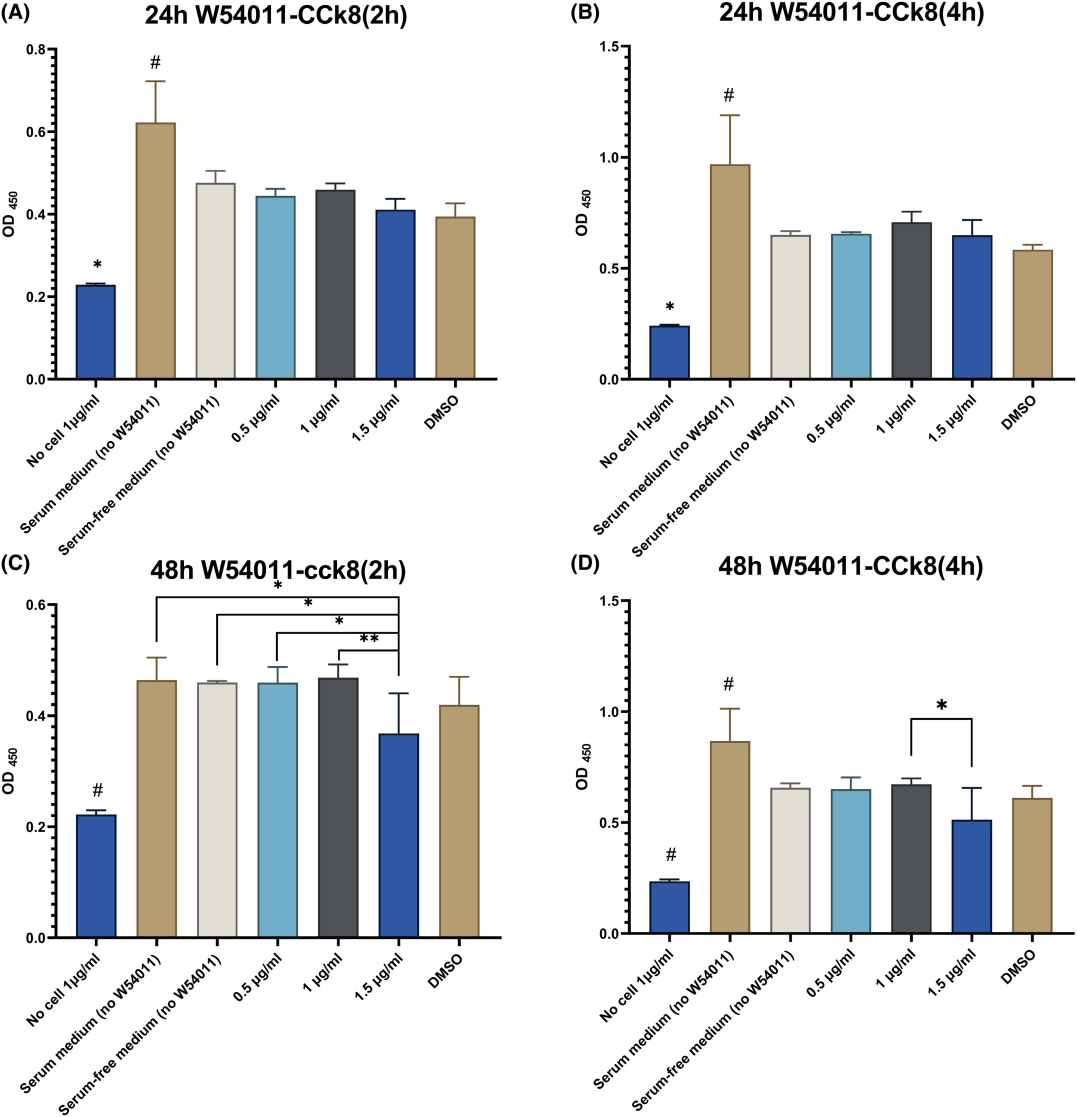
CCK8 cell proliferation assay. Twenty‐four and 48 h after W54011 treated on dental pulp cells. (A) Dental pulp cells treated with different concentrations of W54011 for 24 h were incubated in CCK8 for 2 h; (B) dental pulp cells treated with different concentrations of W54011 for 24 h were incubated in CCK8 for 4 h; (C) dental pulp cells treated with different concentrations of W54011 for 48 h were incubated in CCK8 for 2 h; (D) dental pulp cells treated with different concentrations of W54011 for 48 h were incubated in CCK8 for 4 h. (A, B) *#P* < 0.05 vs. other groups; * *P* < 0.05 vs. other groups. (C) #*P* < 0.05 vs. other groups; *P < 0.05, **P < 0.01 vs. 1.5 μg·mL^−1^ W54011 groups. (D) #*P* < 0.05 vs. other groups; *P < 0.05 vs. 1.5 μg·mL^−1^ W54011 groups. The error bars are the mean with ±SD. Data were analyzed using one‐way ANOVA. *n* = 3.

Human dental pulp cells were cultured to the third generation and 0.5, 1.0 and 1.5 μg·mL^−1^ W54011 were used to detect cell proliferation after 24 and 48 h of treatment (Fig. 2). The cell proliferation efficiency of the three W54011 concentration groups after 24 h was similar to that of the control group (serum‐free DMEM) after 2 and 4 h of CCK‐8 reagent incubation (Fig. 2A,B). However, after 48 h, the 1.5 μg·mL^−1^ W54011 group showed an inhibitory effect on cell proliferation compared with other groups after 2 and 4 h of CCK‐8 reagent incubation (Fig. 2C,D). Moreover, after 48 h, the 1.0 μg·mL^−1^ W54011 group showed similar cell growth compared with the control group (Fig. 2C).

### RT‐PCR analysis

**Fig. 3:**
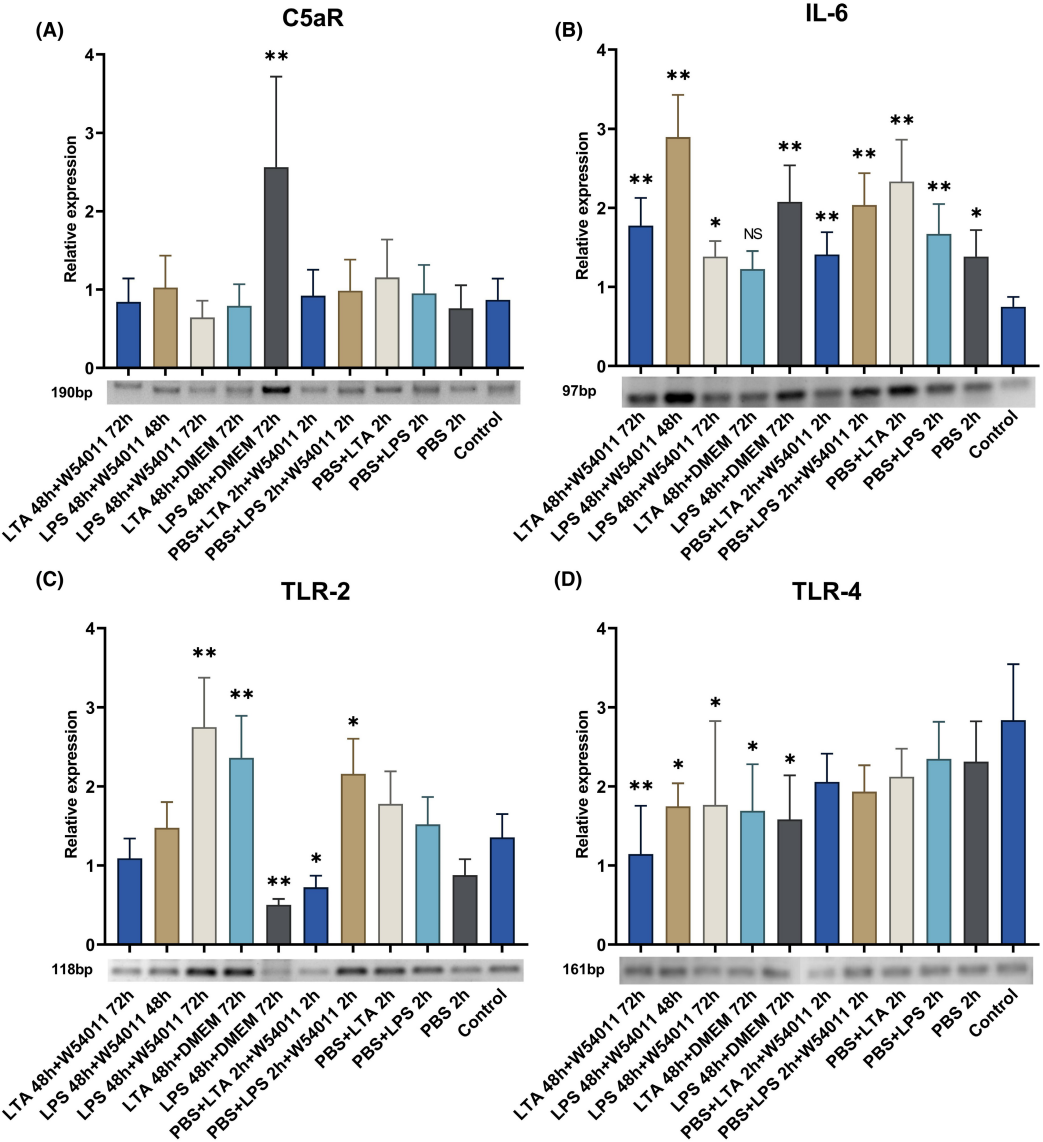
Relative mRNA detection and semiquantitation of C5aR, IL‐6, TLR‐2, and TLR‐4 expression in different groups. Values were normalized to actin expression. The concentrations of LTA or LPS treated for 2 h groups were 10 μg·mL^−1^, other groups were 1.0 μg·mL^−1^. (A) Expression of C5aR in dental pulp cells treated with LPS, LTA, and W54011; (B) expression of IL‐6 in dental pulp cells treated with LPS, LTA, and W54011; (C) expression of TLR‐2 in dental pulp cells treated with LPS, LTA, and W54011; (D) expression of TLR‐4 in dental pulp cells treated with LPS, LTA, and W54011. The error bars are the mean with ±SD (*P < 0.05, **P < 0.01 vs. control group). Data were analyzed using one‐way ANOVA. *n* = 3.

**Fig. 4:**
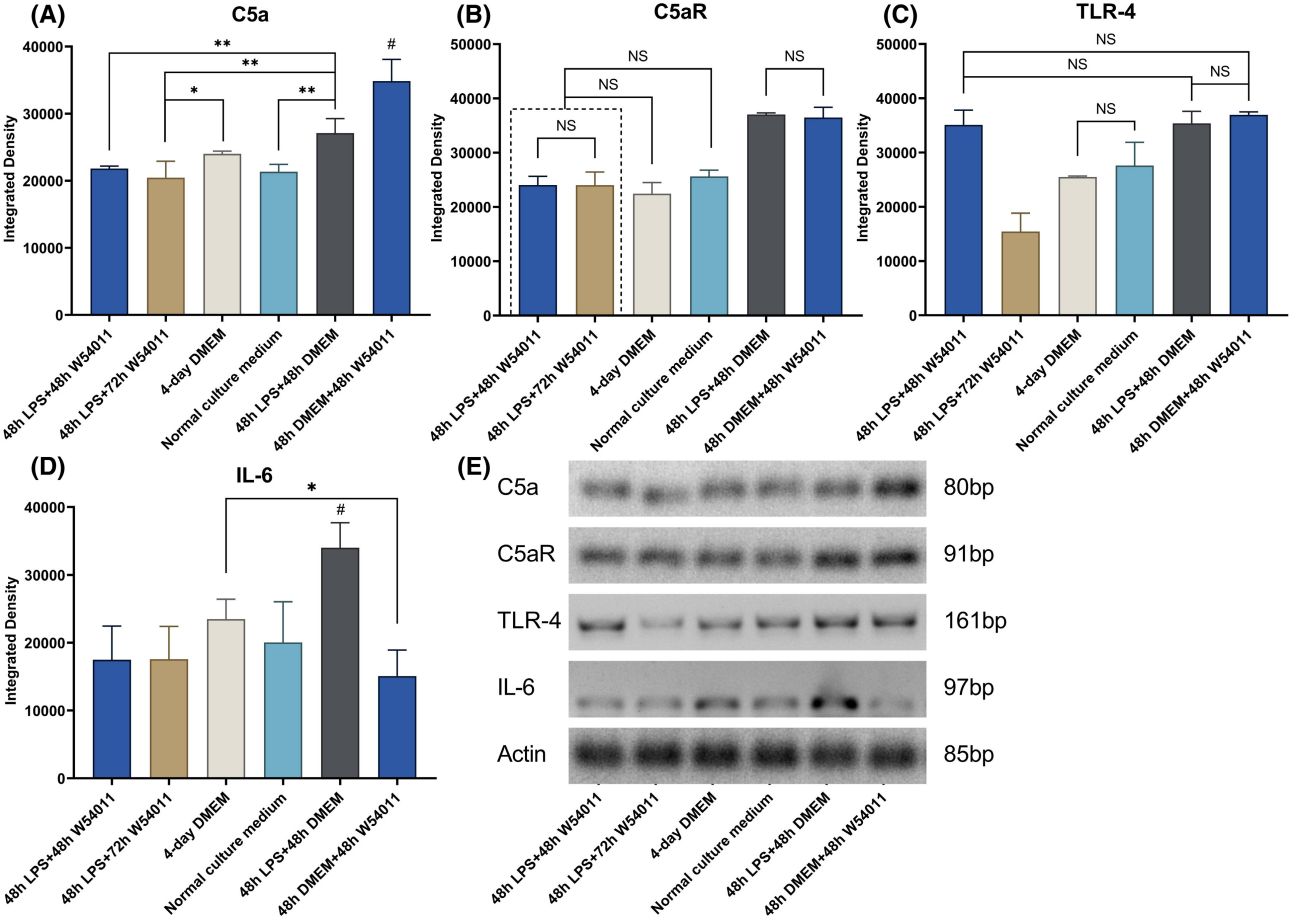
Relative mRNA detection and semiquantitation of C5a, C5aR, IL‐6, and expression in different group. Values were normalized to actin expression. (A) Expression of C5a in dental pulp cells treated with LPS and W54011; (B) expression of C5aR in dental pulp cells treated with LPS and W54011; (C) expression of TLR‐4 in dental pulp cells treated with LPS and W54011; (D) expression of IL‐6 in dental pulp cells treated with LPS and W54011; (E) expression of C5a, C5aR, TLR‐4, IL‐6, and actin. #*P* < 0.05 showed the highest expression of all the groups. *P < 0.05; **P < 0.01; NS means the two groups were not significant. The error bars are the mean with ±SD. Data were analyzed using one‐way ANOVA. *n* = 3.

Following the different treatments, the expression of certain marker genes (i.e., *C5aR*, *IL‐6*, and *TLR‐2*) was evaluated using RT‐PCR analysis (Fig. 3). The expression of *C5aR* was highest in the 48 h LPS stimulation and 3‐day W54011 treatment groups, as reflected in amplicon intensity. Following 48 h of LTA stimulation, the expression of *C5aR* was similar in the groups cultured in DMEM for 72 h and those cultured for 3 days in W54011. The expression of *C5aR* in the groups stimulated by LPS for 2 h was lower than that in the groups stimulated for 48 h (Fig. 3A). The group without LPS stimulation but with W54011 treatment showed the highest *C5a* expression. The 3‐day W54011 treated group showed inhibition of *C5a* expression compared with the LPS‐treated group (Fig. 4A). The group without LPS stimulation but with W54011 treatment and the group with only LPS stimulation showed similar *C5aR* expression levels, which were higher than those of the other groups (Fig. 4B).

Cells in the 48 h LPS stimulation and 3‐day W54011 treatment groups showed lower *IL‐6* expression than those in the LTA group under the same time conditions. Following 2 h stimulation, the expression of *IL‐6* was higher in the LTA group than in the LPS group. Cells in the group treated for 48 h with LTA followed by 3 days with DMEM showed lower *IL‐6* expression than those in the group treated for 48 h with LPS followed by 3 days with DMEM (Fig. 3B). Cells in the group treated with LPS alone showed the highest *IL‐6* expression level. Only cells in the DMEM group showed higher *IL‐6* expression than those in the group without LPS stimulation but with W54011 treatment (Fig. 4D).

Cells in the LPS group showed lower *TLR‐2* expression than those in the LTA group (Fig. 3C); however, after W54011 treatment, the inverse was observed. Moreover, *TLR‐4* expression was significantly reduced in the LPS group after 3 days of W54011 treatment. In the 2 h stimulation groups, regardless of W54011 treatment, no significant differences in *TLR‐4* expression were observed. After LTA or LPS stimulation, cells in the DMEM treatment groups showed lower *TLR‐4* expression (Fig. 3D). There were no differences in *TLR‐4* expression between the group without LPS stimulation but with W54011 treatment, the 2‐day W54011 treatment group, and the group stimulated only with LPS. These three groups showed higher *TLR‐4* expression compared with those of the other groups. The 3‐day W54011 treated group showed the lowest *TLR‐4* expression level. There was no difference in *TLR‐4* expression between the negative control and the 48 h LPS followed by 48 h DMEM groups. Only the DMEM group showed higher *TLR‐4* expression than the negative control, but this level was lower than the other groups except the negative control group (Fig. 4C).

### Quantitative RT‐PCR

**Fig. 5:**
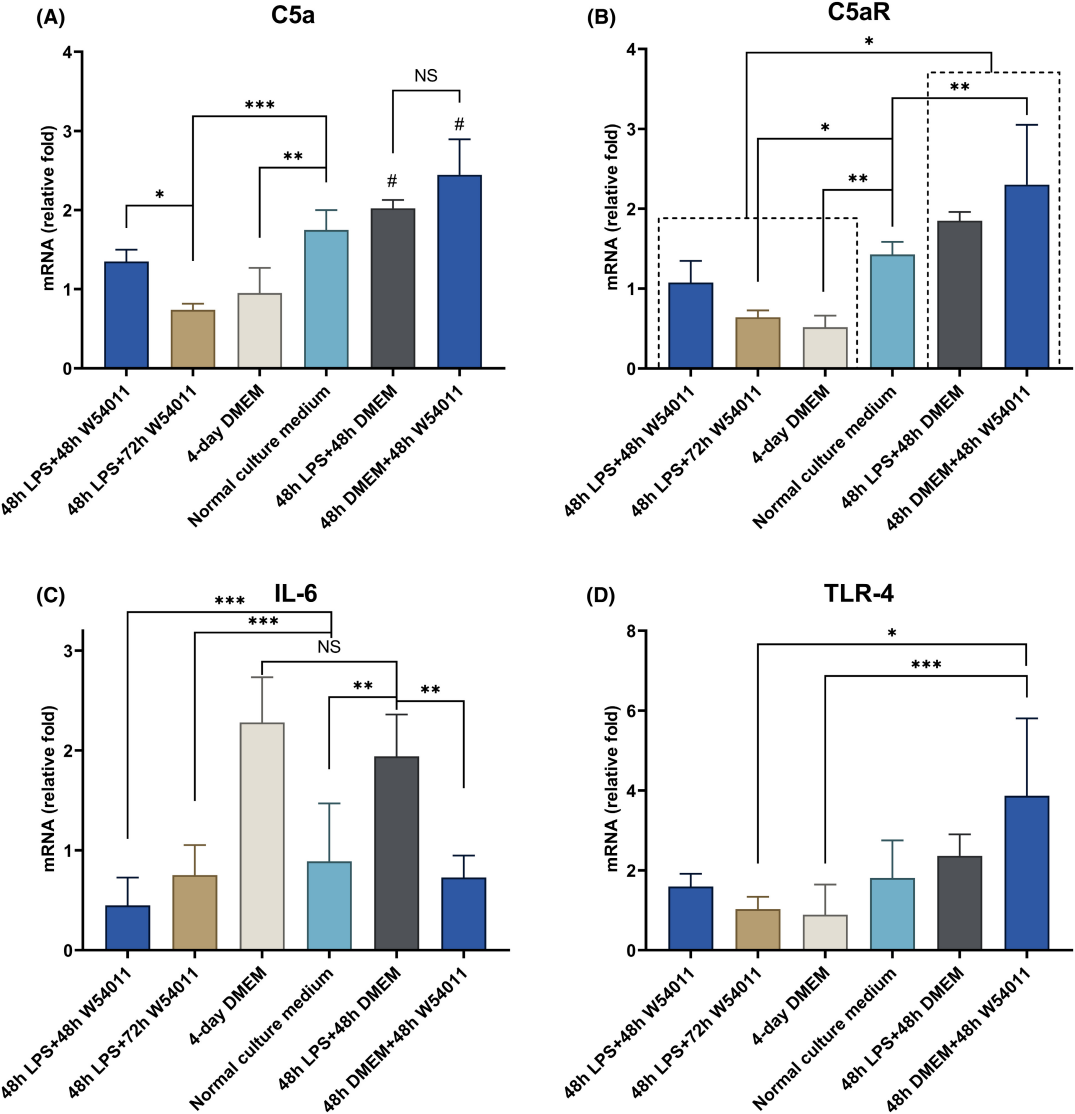
Relative mRNA expression levels of C5a, C5aR, IL‐6, and TLR‐4 by RT‐qPCR. (A) Expression of C5a in dental pulp cells treated with LPS and W54011; (B) expression of C5aR in dental pulp cells treated with LPS and W54011; (C) expression of IL‐6 in dental pulp cells treated with LPS and W54011; (D) expression of TLR‐4 in dental pulp cells treated with LPS and W54011. #*P* < 0.05 compared with other groups;*P <0.05; **P < 0.01; ***P < 0.001. NS means the two groups were not significant. The error bars are the mean with ±SD. Data were analyzed using one‐way ANOVA. *n* = 3.

The inflammatory dental pulp cell models were treated with LPS for 48 h. The 3‐day W54011 treated group showed lower *C5a* expression than the 2‐day W54011 treated group. However, the *C5a* expression in these two groups was lower than that of the untreated group. There was no significant difference in expression between the 2‐day W54011 treated group and the negative control. However, the 3‐day W54011 treated group inhibited *C5a* expression. *C5a* expression in the DMEM alone group was similar to that of the W54011 treated groups. The group without LPS stimulation but with W54011 treatment showed the highest *C5a* expression among all groups. No significant differences in the expression of *C5a* were observed among the 2‐day W54011, 3‐day W54011, and DMEM alone groups. The level of *C5aR* inhibition in the 2‐day W54011 group was similar to that in the negative control group. However, the 3‐day W54011 group showed even lower *C5aR* expression than the negative control group. The group without LPS stimulation but with W54011 treatment showed the highest *C5aR* expression among the groups, except when compared to the LPS‐stimulated group. Only two groups (LPS alone and DMEM alone) showed higher *IL‐6* expression than the other groups. The group with no LPS stimulation but with W54011 treatment showed higher *TL4‐R* expression than the 3‐day W54011‐treated group and DMEM alone group. The other groups showed no significant differences in *TLR‐4* expression (Fig. 5).

### Western blotting

**Fig. 6:**
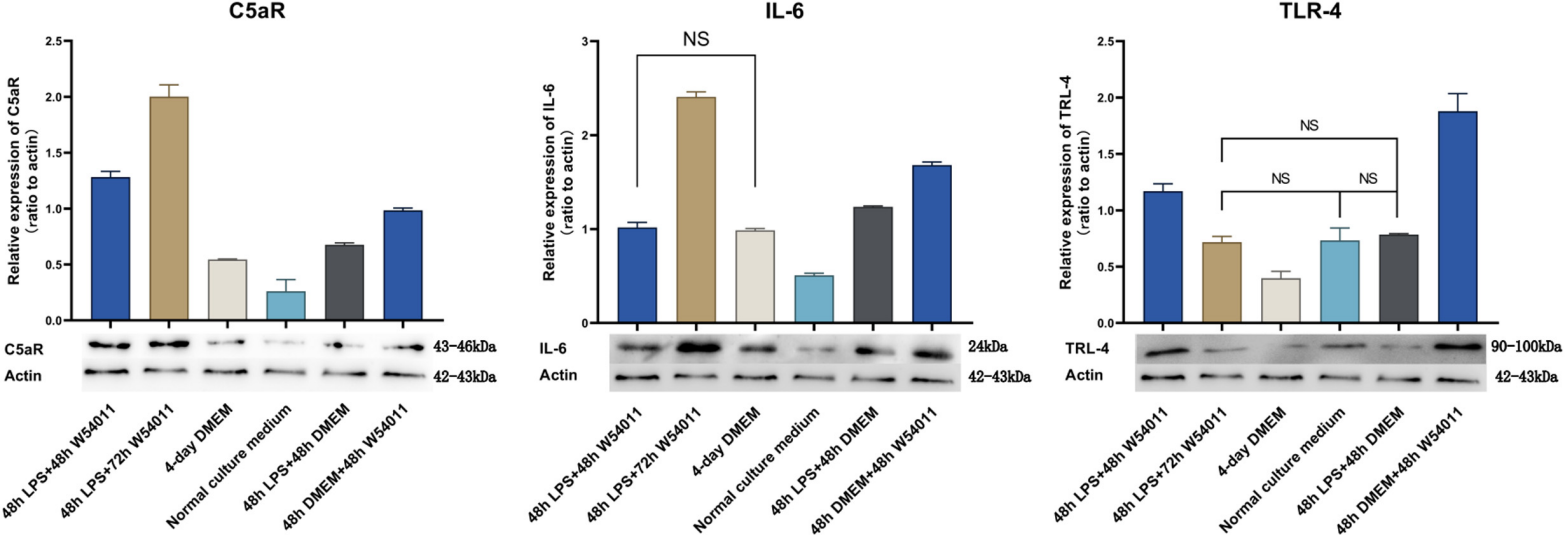
C5aR (43–46 kDa), IL‐6 (24 kDa), TLR‐4 (90–100 kDa), and actin (42–43 kDa) expression detected by western blot analysis. NS means the two groups were not significant. In addition, each of the two groups was statistically significant. The error bars are the mean with ±SD. Data were analyzed using one‐way ANOVA. *n* = 3.

At the protein level, the expression profiles of C5aR and IL‐6 were similar among the different groups. The 2‐day W54011 group showed lower C5aR and IL‐6 expression than the 3‐day W54011 group, which showed the highest expression among all groups. The negative control group showed the lowest C5aR and IL‐6 expression. There were no apparent differences in IL‐6 expression between the 2‐day W54011 group and the DMEM alone group. The 48 h LPS + 2‐day W54011 group showed lower C5aR and IL‐6 expression than the 48 h W54011 group but higher expression than the other four groups. Moreover, the 48 h W54011 group expressed the highest expression of TLR4 than the other four groups (Fig. 6).

## Discussion

Our previous studies have found that *C5aR* expression is significantly upregulated following pulp injury [5]. Blocking *C5aR* expression can significantly inhibit inflammation and promote pulp regeneration. It has been suggested that C5a‐C5aR represents a key node in the balance between immunity and regeneration. Studies in other organs have shown that C5aR is involved in the pathological process of many inflammatory diseases, including cisplatin‐induced glomerulonephritis [17], rheumatoid arthritis, acute lung injury [17], sepsis [18], atherosclerosis, cardiovascular system diseases [19], and tumors [20].

W54011 is an orally active, nonpeptide, complement C5a receptor antagonist that is undergoing preclinical investigation with Mitsubishi Pharma Co. (Osaka, Japan) W54011 inhibits intracellular Ca2þ mobilization, chemotaxis, and the production of reactive oxygen species [21]. Moreover, it can effectively inhibit the binding of C5a‐C5aR and inhibit the expression of inflammatory factors. Several studies have used W54011 to treat peripheral neuropathies [22] and C5a‐induced neutropenia [21]. Moreover, it can protect lung cells and tissues against LPS‐induced injury [23] and can be used to intervene in mouse periodontitis [24]. Whether W54011 affects the expression of TLR‐2/4 and the inflammatory factor IL‐6 by inhibiting the binding of C5a and C5aR is still inconclusive. Moreover, it is worth studying the action time and concentration of C5sR antagonists in the treatment of dental pulp cells. Here, we sought to block the C5a‐C5aR axis using W54011 to find new therapeutic targets in the treatment of deep caries that approaches the pulp.

Several studies have used LTA‐ or LPS‐stimulated dental pulp cells to generate inflammation models. LPS first binds to its binding protein (lipopolysaccharide‐binding protein, LBP) and then to lipopolysaccharide (CD14), which is fixed on the cell surface through glycosylphosphatidylinositol (GPI). LPS activates TLR‐4 signal transduction as an LPS‐LBP‐CD14 trimeric complex. TLR‐4 enables cells to recognize LPS. The NF‐κB signal transduction pathway is the most important downstream pathway in the signal transduction pathway mediated by LPS. Active NF‐kB migrates to the nucleus and induces the expression of various inflammation‐related specific genes, such as TNF, IL‐6, and IL‐8. Therefore, we tested the expression of *TLR‐4* and studied the effect of W54011 on the inflammatory stimulation of dental pulp cells. C5a is a protein that frees to the liquid phase after C5 cleavage, and its expression cannot be detected by western blot. More targeted inhibition can be achieved by targeting the major byproducts of C5 cleavage, such as C5a and its targets, C5a receptors. In previous studies, in addition to 48 h of LTA and LPS stimulation [5], some studies used 2 h to stimulate cells [11, 12]. The concentration of LTA or LPS was 10 or 1 μg·mL^−1^. Our previous report compared the expression changes of LPS stimulation from 24 h to 7 days, and the results showed that after 48 h of LPS and LTA stimulation, a dental pulp cell inflammation model with high *IL‐6* expression can be obtained [5]. Therefore, in this study, we first set up a grouping situation with both 48 and 2 h stimulation. The semiquantitative PCR results clearly showed differences in the amplicons of *C5aR*, *TLR‐2*, *TLR‐4*, and *IL‐6* after stimulation with LPS and LTA at different times and concentrations.

Gram‐negative bacteria are more pathogenic. In cases of deep caries that approach the pulp, some scholars measured the proportion of Gram‐negative and Gram‐positive bacteria and found that Gram‐negative bacteria accounted for a higher proportion in the cases of symptomatic deep caries that approach the pulp. The expression of *IL‐6* and *C5aR* confirmed that the inflammatory reaction of LTA was faster than that of LPS, while LPS has a stronger inflammatory effect than LTA. Moreover, the optimal concentration and duration of action of the W54011 antagonist were determined to be 1 μg·mL^−1^ LPS stimulation for 48 h and 1 μg·mL^−1^ W54011 for 48 h.

Thus far, no studies have looked at whether W54011 can be used to treat dental pulp‐related inflammation. One study demonstrated the link between inflammation and dental tissue regeneration through complement activation [25]. Another revealed a key role for the complement C5a receptor in dental pulp cell odontolineage differentiation and *in vivo* dentin regeneration [26]. However, in this study, they only cultured the dental pulp cells with W54011 (10 nmol·L^−1^) or the C5aR agonist (C5a, 20 nmol·L^−1^) for 72 h. Here, we studied the effect of W54011 alone, and in combination with LPS stimulation. Another study revealed the roles of C5aR and inflammation in modulating brain‐derived neurotrophic factor (BDNF) and nerve growth factor (NGF) in dental pulp cells [27]. However, the stimulus condition was different from our study. First, W54011 10 nmol·L^−1^ equaled 5 × 10^−6^ μg·mL^−1^, which was much lower than the concentration we selected for our study (1 μg·mL^−1^). They stimulated dental pulp cells with LPS (1 μg·mL^−1^) for 1 h, which was much shorter than our stimulus duration (48 h). There are currently no relevant articles on the exact recommended concentration and duration of action of W54011 to cure inflammatory dental pulp cells. After a series of experiments, we recommended that 1 μg·mL^−1^ LPS stimulation for 48 h and treatment with 1 μg·mL^−1^ W54011 for 48 h can effectively inhibit inflammation of dental pulp cells. In a previous article, we described the promotion of C5a on the differentiation of dental pulp cells into odontoblasts [28], which was confirmed by other studies [29]. Therefore, we chose W54011 to inhibit the inflammation. As a nonpeptide competitive antagonist, W54011 will not affect the expression of C5a or block the immune system. However, competitively binding to C5aR effectively inhibits the formation of C5a‐C5aR, which in turn hinders the immune process.

Quantitative PCR showed that in the 2‐day W54011 group, the inhibitory effect of W54011 on C5aR and the expression of IL‐6 were the same as those in the negative control group. It also showed that the inhibitory effect on C5aR at the 3‐day time point was good. However, the 3‐day W54011 group had an inhibitory effect on C5a. Interestingly, only the W54011 group expressed high levels of C5a, C5aR, and TLR‐4. There was no inhibiting effect of 2‐day W54011 compared with the negative control group in the protein expression level. However, the purpose of this study was to compare the inhibitory effects of W54011 on C5aR and IL‐6 expression at different time points. The 48 h W54011 treatment group expressed lower C5aR and IL‐6 compared with the 72‐h W54011 treatment group. Therefore, W54011 may have an initiating signal effect. Further research is needed to clarify the exact cause and mechanism of W54011.

We selected W54011 to inhibit the C5a‐C5aR axis to effectively inhibit IL‐6 expression. Additionally, we wanted to obtain as much C5a expression as possible, which is conducive to the differentiation of teeth. To avoid an influence on the expression of C5a, we selected 2 days of W54011 on dental pulp cells after 48 h of LPS stimulation.

Therefore, this study concluded that LPS stimulation for 48 h, followed by treatment with W54011 for 48 h, can effectively inhibit inflammation without affecting C5a expression. Compared with other stimulus conditions, this study provides an important scientific basis for follow‐up studies of W54011 in dental pulp cells.

## Conflict of interest

The authors declare no conflict of interest.

## Author contributions

JH and ML conceived of and designed the study, performed the experiments, and wrote the manuscript. XT and XW participated, carried out experiments, and made substantial contributions to the acquisition of data and were involved in the drafting of the manuscript. LG and WH performed the surgeries to obtain teeth and were involved in revising the manuscript. NL, ZJ, XC, and HY organized the images and were responsible for data curation and formal analysis. LT and ML helped write the manuscript, acquired funding, conceived of and designed the study, and provided the final approval of the manuscript for publication. All authors reviewed the manuscript.

## Data Availability

The datasets used in the current study are available from the corresponding author upon reasonable request.
