# Corrigendum to: Determination of the optimal concentration and duration of C5aR antagonist application in an inflammatory model of human dental pulp cells

**DOI:** 10.1002/2211-5463.13712

**Published:** 2023-10-12

Hu, J., Tan, X., Wei, X., Hu, W., Gao, L., Cao, X., Yang, H., Jiang, Z., Li, N., Teng, L. and Liu, M. (2023), Determination of the optimal concentration and duration of C5aR antagonist application in an inflammatory model of human dental pulp cells. *FEBS Open Bio*, 13: 570–581. https://doi.org/10.1002/2211-5463.13571

The original article contained errors in Fig. 3D: the mRNA gel contained an undeclared splice site, and the presented quantifications were incorrect. In addition, the original Fig. 3 did not show the actin bands used for normalization. A corrected version of Fig. 3 is shown below. These errors do not affect the conclusions of the article.

**Fig. 3:**
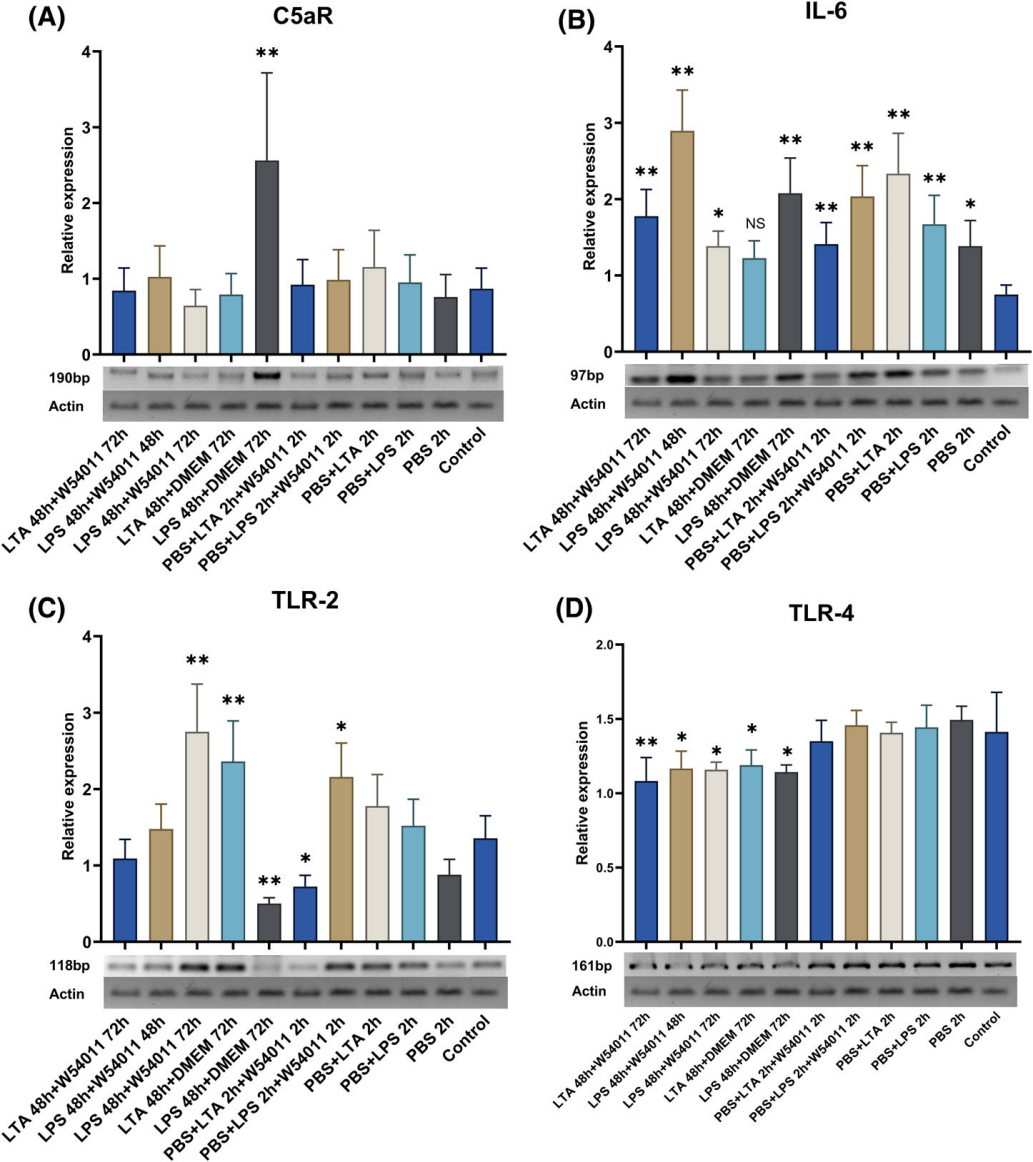
Relative mRNA detection and semiquantitation of C5aR, IL‐6, TLR‐2, and TLR‐4 expression in different groups. Values were normalized to actin expression. The concentrations of LTA or LPS treated for 2‐h groups were 10 μg·mL^−1^, and other groups were 1.0 μg·mL^−1^. (A) Expression of C5aR in dental pulp cells treated with LPS, LTA, and W54011; (B) expression of IL‐6 in dental pulp cells treated with LPS, LTA, and W54011; (C) expression of TLR‐2 in dental pulp cells treated with LPS, LTA, and W54011; (D) expression of TLR‐4 in dental pulp cells treated with LPS, LTA, and W54011. The error bars are the mean with ±SD (*P < 0.05, **P < 0.01 vs. control group). Data were analyzed using one‐way ANOVA. *n* = 3.

